# Open-source software for respiratory rate estimation using single-lead electrocardiograms

**DOI:** 10.1038/s41598-023-50470-0

**Published:** 2024-01-02

**Authors:** Jesse D. Roberts, Richard D. Walton, Virginie Loyer, Olivier Bernus, Kanchan Kulkarni

**Affiliations:** 1 Departments of Anesthesia, Pediatrics, and Medicine, Massachusetts General Hospital, Boston, MA USA; 2 IHU-LIRYC, Heart Rhythm Disease Institute, Fondation Bordeaux Université, 33600 Pessac, Bordeaux, France; 3 INSERM, Centre de Recherche Cardio-Thoracique de Bordeaux, U1045, University of Bordeaux, 33000 Bordeaux, France

**Keywords:** Biomedical engineering, Respiration

## Abstract

Respiratory rate (RR) is a critical vital sign used to assess pulmonary function. Currently, RR estimating instrumentation is specialized and bulky, therefore unsuitable for remote health monitoring. Previously, RR was estimated using proprietary software that extract surface electrocardiogram (ECG) waveform features obtained at several thoracic locations. However, developing a non-proprietary method that uses minimal ECG leads, generally available from mobile cardiac monitors is highly desirable. Here, we introduce an open-source and well-documented Python-based algorithm that estimates RR requiring only single-stream ECG signals. The algorithm was first developed using ECGs from awake, spontaneously breathing adult human subjects. The algorithm-estimated RRs exhibited close linear correlation to the subjects’ true RR values demonstrating an R^2^ of 0.9092 and root mean square error of 2.2 bpm. The algorithm robustness was then tested using ECGs generated by the ischemic hearts of anesthetized, mechanically ventilated sheep. Although the ECG waveforms during ischemia exhibited severe morphologic changes, the algorithm-determined RRs exhibited high fidelity with a resolution of 1 bpm, an absolute error of 0.07 ± 0.07 bpm, and a relative error of 0.67 ± 0.64%. This optimized Python-based RR estimation technique will likely be widely adapted for remote lung function assessment in patients with cardiopulmonary disease.

## Introduction

Respiratory rate (RR) is a critical vital sign. The accurate measurement of RR is essential for assessing the pulmonary status of patients with lung and respiratory abnormalities, such as chronic obstructive pulmonary disease, restrictive lung disease, sleep apnea, and Cheyne-Stokes respiration^1–4^. Monitoring the RR of critically ill patients in the hospital also plays a significant role during mechanical ventilation. This can detect inadvertent disconnection from the ventilator gas circuit and, together with a measurement of tidal volume, helps assess whether adequate ventilation is provided while minimizing lung trauma^5^. RR is an important parameter that is used to assess the clinical status of patients of all ages, even newborns^6,7^. With the increasing need for ambulatory telehealth systems^8,9^, the remote assessment of RR is a critical factor that will enable the expanded monitoring of outpatients for pulmonary disease assessment and management.

Because of the importance of respiratory monitoring, novel methods for estimating RR using non-contact- and contact-based modalities have been explored over the past two decades^10^. Contactless RR monitoring, which may entail radar, optical, or thermal sensors, offers promising new opportunities. These monitoring methods might be especially useful for patients whose contact-based monitoring is technically difficult, uncomfortable, or considered cumbersome and, hence, associated with poor compliance. However, devices employing these technologies are not broadly used. This is because the clinical applicability and utility of their underlying methods have yet to be completely determined. Contact-based RR measurement methods are more clinically developed. These methods estimate RR by measuring respiratory sounds, carbon dioxide levels in exhaled gases, respiratory gas flow, or changes in blood oxygen saturation (SpO_2_). In the latter case, pulse oximetry evaluates the SpO_2_ levels using photoplethymography, quantifying changes in the optical absorption properties of hemoglobin with oscillations in blood flow^10,11^. The pulsatile photophlethysmogram captured with these sensors has been used to extract respiratory and pulse rate in addition to hemoglobin oxygen saturation. Currently, contact-based respiratory inductive plethysmography is the most accepted gold standard methodology used to estimate RRs. However, this method involves the use of multiple sensor-bands, spread across the chest, to quantify rib cage and abdominal movement. Furthermore, the bands incorporate electrical wires, which need to be excited by low amplitude, high frequency alternating current. This increases the complexity and expense of the equipment required to estimate RR using this method. These factors limit the adaptability of this technology for remote monitoring. Specialized technologies that use some of these non-contact- and contact-based technologies are already available in commercial devices that enable real-time RR monitoring (recently reviewed in^11^). Yet, many of these devices depend on pulse oximetry or photoplethysmography to estimate RR, and reports suggest that the accuracy and precision of these devices for continuous RR monitoring have room for improvement^11–13^. Previous studies report RR differences ranging from 2–6 bpm of the actual values^14^ with estimation errors as high as 8–10%^15–17^. Newer contact-based methods employing fiber optics, flowmeters, acoustics or thermal sensors have varying ease of utility, and have demonstrated errors ranging from 2–10% (up to 4 bpm), with generally higher estimation errors during walking or running^18^. In addition, during a comparative evaluation of over 253 algorithms, Charlton and colleagues reported that only 36.4% algorithms were more precise when using photoplethysmography as compared to electrocardiogram (ECG) data^12^.

ECG-derived RR measurement is being developed as a new contact-based monitoring technique. Broadly, this method estimates RR using algorithms that model and quantify modulations in ECG voltage waveform morphology that are induced by chest movements during breathing. Several ECG-derived RR estimation algorithms have been proposed recently. These are primarily based on modeling the oscillations in QRS waveforms obtained from ECG signals that are gathered from several sensors arrayed over the subject's chest or by using these signals to estimate deviations in the axis of the heart caused by respiration^8,19–25^. Moody et al. were among the first to demonstrate the use of multi-lead ECG waveforms to estimate RR based on the deflections in the cardiac axis^24^. To offset the need for orthogonal ECG lead selection, which is necessary for calculating the angle of the cardiac axis, Weiss et al.^26^ developed an algorithm that identified the optimal ECG lead combination for RR estimation independent of the orientation of the leads. Their method iteratively calculated the ratios of the envelope of QRS amplitudes from each lead combination and determined the optimal pairs for RR estimation based on the largest spectral signal-to-noise ratio. Later, other groups reported algorithms for ECG-derived RR estimation based on determining such QRS amplitude modulations^19–21,23^. Although these methods require analysis of ECG signals from several leads, Khaled and coworkers derived RR estimations from single-lead ECG signals using variations in R-peak amplitude alone^23^. Importantly, a quantitative comparison of the available methods has suggested that RR estimates from single-stream ECG signals might be more robust than those based on estimating cardiac axis deviations using multi-lead ECG data^22^. In addition, a comprehensive evaluation of just over 300 RR estimation algorithms by Charlton et al. suggested that time-domain-based RR estimation techniques might perform better than most frequency domain or photoplethysmography-based detection techniques^12^. This was found especially when these methods were combined with a smart fusion technique^27^, which weighs RR estimates based on the modulation of respiratory factors, including baseline wander and amplitude and frequency modulation^12^. However, it is unknown whether a combination of time and frequency-domain-based modeling of ECG from a single-lead ECG waveform can produce accurate RR estimation.

In this current work, we introduce an open-source, Python script-based algorithm that estimates RR by extracting time and frequency domain modulations from a single stream of ECG signal. First, ECG data from spontaneously breathing human subjects performing a series of physical tasks were used to refine and test the algorithm and to demonstrate its potential clinical relevance. Then, data from sheep with evolving myocardial ischemia and infarction (MI) were used to demonstrate the robustness of the RR estimation. Specifically, the algorithm efficacy was tested using the associated abnormal ECG waveforms when heart disease modifies its electrical activity. This work offers an approachable and computationally efficient alternative to the currently available RR estimation methods for patient RR monitoring. The algorithm is anticipated to be integrated into lightweight ECG sensor systems to enable remote monitoring of respiratory function.

## Results

### Validation of RR estimation using spontaneously breathing healthy human subjects

**Figure 1:**
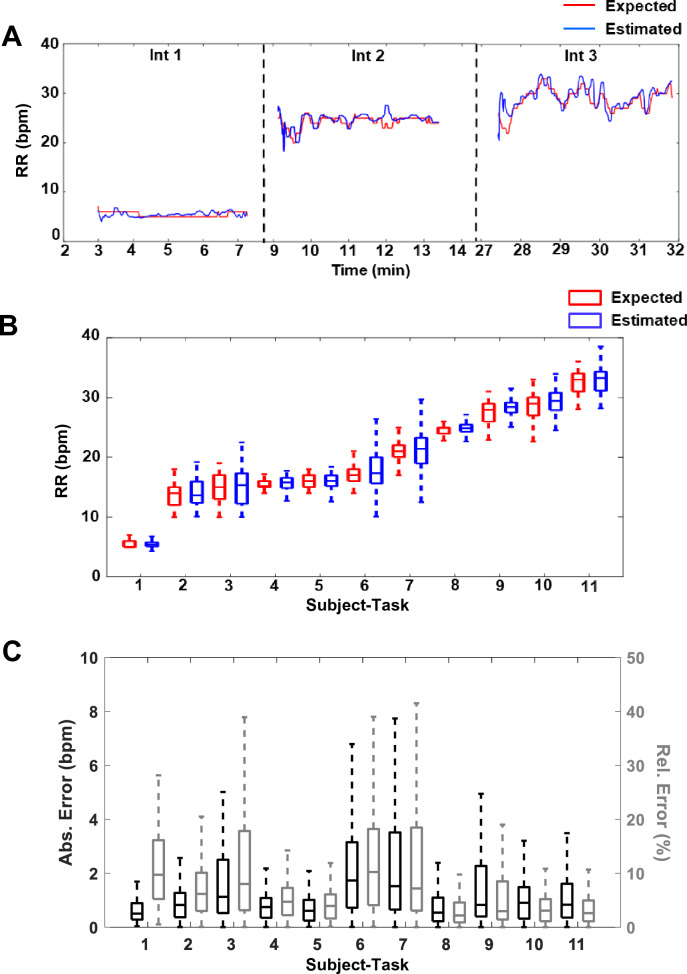

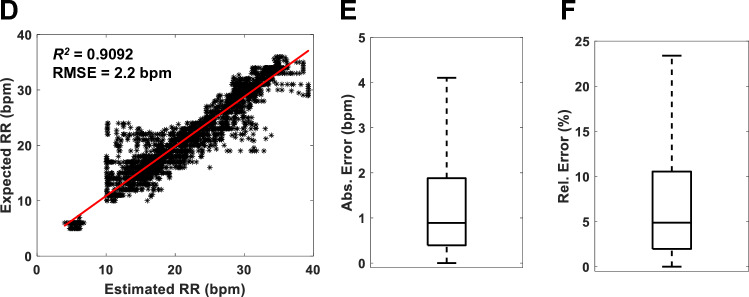
Respiratory rate (RR) estimation in spontaneously breathing humans. (**A**) RR estimates, in breaths per minute (bpm), during 3 levels of exercise in one subject. Algorithm-estimated RRs (estimated, blue) are compared with RR measured from the subject using the respiratory inductive plethysmography based Hexoskin monitor (expected, red) while performing three consecutive tasks: (1) resting, standing upright on a treadmill (Int 1); (2) walking on the treadmill at a moderate speed (1.2 m/s) (int 2); and (3) walking on the treadmill with 15% track inclination at the moderate speed (Int 3). (**B**) Summary results of algorithm-estimated and reference RRs (blue and red, respectively) during each subject-task interval. The data are from seven subjects, each performing either or all the three levels of exercise described above (subject-tasks), and presented in order of increasing average expected RR values. (**C**) The absolute errors (black) and relative errors (gray) of the algorithmic RR estimations across the subject-task intervals described above. Equivalence testing revealed that the expected and estimated RRs were the same (*p* < 0.0001) for all subject-task intervals. (**D**) A comparison of the ECG cycle-to-cycle estimated and expected RR for all subjects and tasks with the indicated R^2^ value (0.9092) and low root mean square error (RMSE, 2.2bpm) support a close linear relationship between the values. (**E**) Absolute error (bpm) and (**F**) relative error (%) distributions across all subjects.

We developed an algorithm that estimates RR by quantifying breath-induced oscillations in the envelope of the ECG QRS complexes from a single lead. The details about its development and implementation as an open-source script in Python are provided in *Methods*. We tested the algorithm using paired ECG and RR data generated previously by others^28,29^ and obtained from an open-source repository^28^. Representative data consisting of the reference RR determined using the respiratory inductive plethysmographic method (Expected, red), and RR estimated algorithmically using our method (Estimated, blue) from one subject are depicted in Fig. 1A. These data suggest that the RR estimates closely and rapidly track those determined using the respiratory inductive plethysmography method. Of note, the RRs determined by both methods are not constant but vary during each task interval; this is expected because the patients are breathing of their own volition, spontaneously throughout them. A close correspondence between the plethysmographic and algorithmic RRs estimations was also detected for each experimental intervention across all subjects, as depicted in Fig. 1B. Importantly, equivalence testing revealed that across the range of RRs, those detected algorithmically were identical to those determined by the biosensor device (*p* < 0.0001). A fit performed between the instantaneous cycle-to-cycle algorithm-estimated and reference RRs during the tasks demonstrated a high linear correlation and low variance, with an R^2^ of 0.9092 (Fig. 1D) and root mean square error of 2.2 bpm. The estimated RRs demonstrated low absolute and relative errors during each task across all subjects (Fig. 1C), with a median absolute RR estimation error of 0.89 ± 1.82 bpm (Fig. 1E) and a median relative estimation error of 4.88 ± 9.47% (Fig. 1F) of the RR.

### Validation of algorithmic RR estimation when ECG morphology is altered by myocardial ischemia and infarction in sheep

We tested the effect of electrophysiological changes induced by myocardial ischemia and MI on the accuracy of RR determination. An anesthetized, mechanically ventilated chronic MI ovine model was used for this work, which we have previously demonstrated to precisely replicate the changes in ECG QRS morphology observed in humans with ischemic heart disease^30^. The changes in heart rate, QTc, and ST height observed in the sham and MI groups at the three time points, baseline, myocardial ischemia just after coronary occlusion, and MI, are summarized in Table 1. The heart rate and QTc interval significantly increased after six weeks of MI, a phenomenon that was absent in the sheep without myocardial ischemia and infarction that underwent a sham procedure. Also, the significant elevation in ECG ST segment height observed with acute ischemia (post coronary artery occlusion) returned to baseline levels after six weeks of myocardial remodeling. Similar phenomena of non-persistent ST elevation can be observed in some humans after myocardial ischemia^31^.

**Table 1:** Summary results of the sheep cardiac parameters. Data are presented as means ± standard deviation. * and ^†^ indicate a difference between the paired results. The respective *p*-values are as follows: for heart rate comparisons, *p = 0.0414 and ^†^*p* = 0.0043; for QTc comparisons, *p = 0.00045 and ^†^*p* = 0.001; for ST height comparisons, *p = 0.0048 and ^†^*p* = 0.0015.

Parameter	Sham			Chronic MI		
	Baseline	Post Occ	Chronic MI	Baseline	Post Occ	Chronic MI
Heart Rate (beats/min)	71.01 ± 16.90	67.00 ± 11.89	76.26 ± 9.12	69.33 ± 13.29*	64.12 ± 10.01^†^	80.08 ± 13.47*^†^
QTc (seconds)	0.387 ± 0.032	0.408 ± 0.020	0.439 ± 0.035	0.377 ± 0.040*	0.385 ± 0.043^†^	0.464 ± 0.049*^†^
ST height (mV)	0.023 ± 0.018	0.014 ± 0.009	0.009 ± 0.006	0.017 ± 0.012*	0.065 ± 0.047*^†^	0.012 ± 0.009^†^

**Figure 2:**
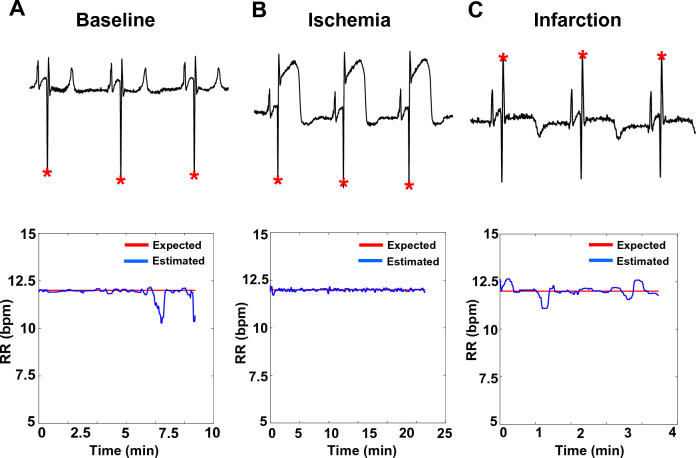
ECG and RR estimation during chronic MI in sheep. Top panel: Representative ECG traces are depicted during baseline (**A**), post coronary artery occlusion during acute myocardial ischemia (**B**), and after six weeks when chronic myocardial infarction has developed (**C**). The red symbols mark the algorithm-detected R-peaks. Bottom panel: Estimated RR of a representative sheep mechanically ventilated at the indicated RR (in breaths per minute, bpm) for the indicated time during the three periods described above.

**Figure 3:**
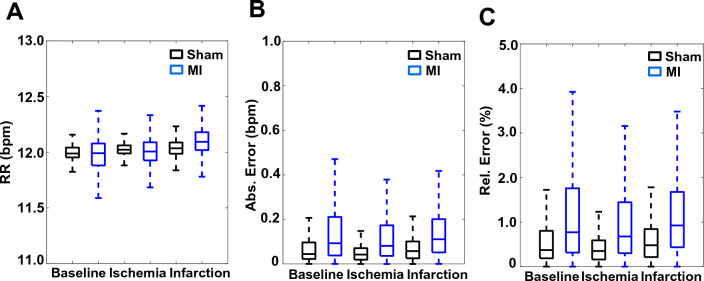
RR estimation in sheep with chronic MI disease. (**A**) Estimated RRs (breaths per min, bpm) in sham (n = 5) and chronic MI (n = 13) sheep during the baseline, ischemia, and infarction phases of the study. (**B**) and (**C**) are the absolute and relative estimation errors, respectively, of the data illustrated in (**A**). Equivalence testing revealed that the estimated RRs between the sham and MI groups at all time periods were the same (*p* < 0.0001).

Figure 2 shows ECG traces from a representative anesthetized and mechanically ventilated sheep before the intervention (Baseline) and associated with myocardial ischemia and infarction, as labeled. As depicted in the figure, there was a close correspondence between the ventilator-delivered and the estimated RR. Importantly, the changes in ECG morphology during acute ischemia and progression to infarction did not affect the algorithm's accuracy. The high level of algorithm performance was also detected with the larger numbers of sheep, including those with cardiac ischemia, infarction, or who underwent sham procedures (Fig. 3). Importantly, equivalence testing revealed that the RRs determined algorithmically were identical to those delivered by the mechanical ventilator despite the changes in ECG morphology (*p* < 0.0001). The estimated RR, along with the absolute and relative RR estimation errors observed in the sham and MI groups at the 3-time periods, are summarized in Table 2.

**Table 2:** Summary results of the sheep estimated respiration rate, absolute error, and relative error at a ventilator respiratory rate setting of 12 bpm.

Parameter	Sham			Chronic MI		
	Baseline	Post Occ	Chronic MI	Baseline	Post Occ	Chronic MI
RR (bpm)	11.48 ± 1.93	11.92 ± 0.94	11.97 ± 0.63	11.48 ± 1.78	12.22 ± 1.96	12.06 ± 0.78
Abs. error (bpm)	0.56 ± 1.91	0.15 ± 0.93	0.13 ± 0.62	0.65 ± 1.73	0.51 ± 1.91	0.23 ± 0.75
Relative error (%)	4.71 ± 15.95	1.26 ± 7.79	1.11 ± 5.14	5.45 ± 14.44	4.26 ± 15.89	1.90 ± 6.21

**Figure 4:**
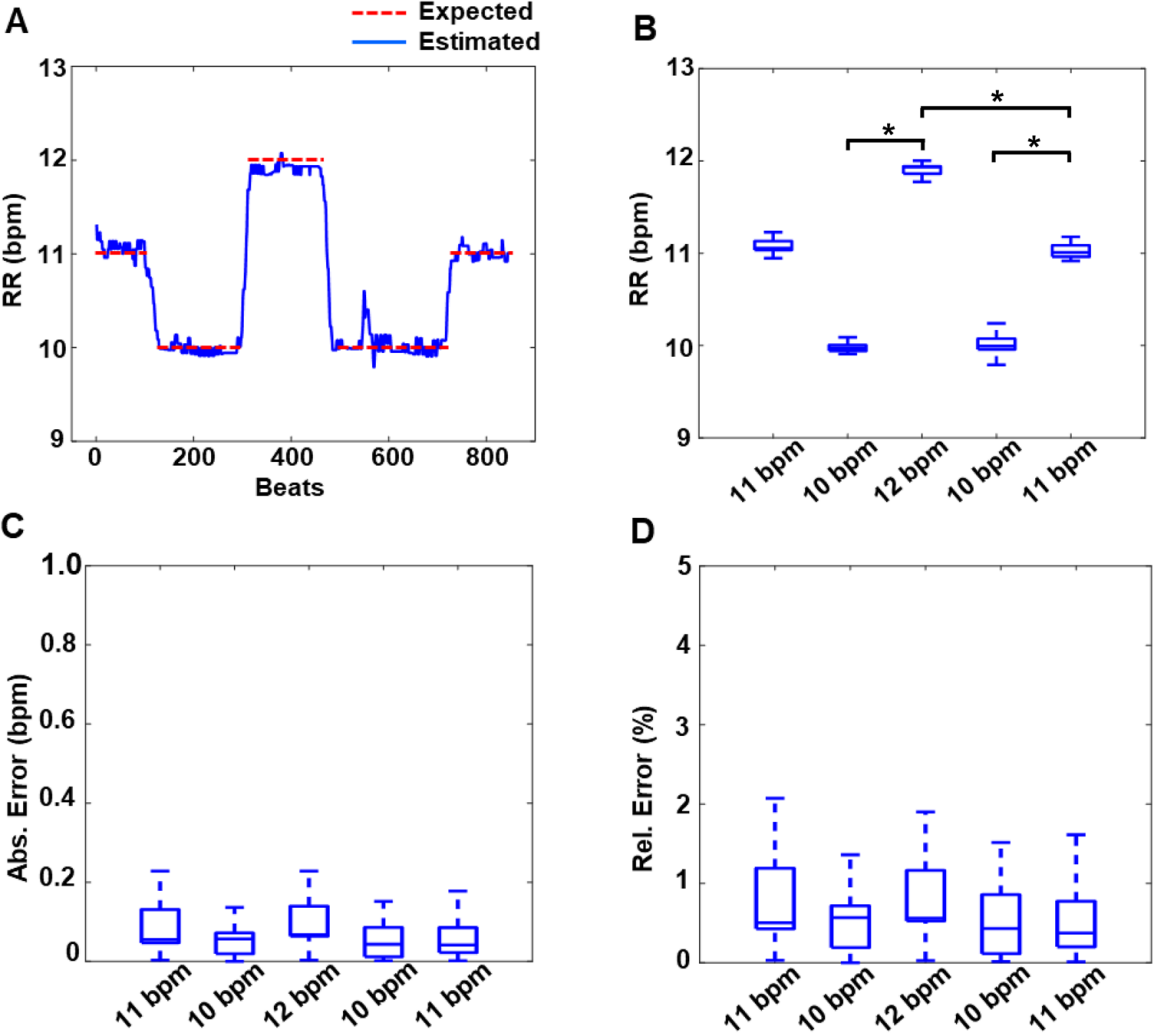
Precision of RR estimation in sheep with chronic MI during changing respiration rates. (**A**) Instantaneous RR estimates, in breaths per min (bpm), of a representative sheep during small successive changes in mechanical ventilation rates. (**B**) Boxplot of instantaneous RR estimates during the indicated sequential ventilator RR changes. The RR estimates at the ventilator settings were significantly different from each other (*p < 0.0001). (**C**) and (**D**) Absolute and relative error during the indicated successive RR setting changes. (Panels A-D, n = 1 sheep).

As shown in Fig. 4A, using single-lead ECG data obtained from a representative sheep (sham) the algorithm quickly detects even small changes in RR when the ventilator breathing rates are varied. The high precision in RR estimation is supported by our detecting a small standard deviation in the calculated RR in the single sheep with the ventilator changes (Fig. 4B). The corresponding absolute and relative estimated RR errors are summarized in Fig. 4C and D. The estimated RR at each setting was significantly different from every other setting (10.01 ± 0.10 bpm vs. 11.04 ± 0.07 bpm, *p* < 0.0001; 10.01 ± 0.1 bpm vs. 11.91 ± 0.05 bpm, *p* < 0.0001; and 11.04 ± 0.07 bpm vs. 11.91 ± 0.05 bpm, *p* < 0.0001), denoting the efficiency of the algorithm in distinguishing between RRs with a precision of 1 bpm. The mean absolute RR estimation error was 0.07 ± 0.07 bpm, and the mean relative estimation error was 0.67 ± 0.64% across all RR settings.

## Discussion

RR monitoring is critical in patients with cardio-respiratory diseases. This is because changes in RR can be associated with evolving cardiopulmonary disease and help dictate the responses to therapies. While many non-invasive techniques have been promoted for estimating RR, there is a need for efficient, scalable, and portable respiratory monitoring devices that can enable remote tracking of this important vital sign in hospital and outpatient settings. To facilitate such monitoring, we have developed and tested a simplified open-source Python script-based algorithm that we demonstrate can estimate RR from single-lead ECG signals. The main findings of our study are that: (1) clinically relevant RR estimates can be extracted using single-lead body surface ECG from human subjects; (2) ECG morphology associated with cardiac ischemia and infarction does not compromise algorithm accuracy; and (3) the algorithm rapidly tracks even small changes in RR with high accuracy and precision.

Currently, respiratory inductive plethysmography and impedance pneumography remain the most commonly used method for clinically monitoring RR^32,33^. However, these methods require cumbersome equipment that estimates RR based on the use of sensors that detect chest movements, and this decreases their utility in remote monitoring applications. ECG-derived RR estimators offer an attractive solution for continuous RR monitoring primarily due to their employment of lightweight, battery-powered ECG monitors. They don’t require specialized RR-measuring equipment, which makes them easy to use, and subjects can use simple ECG electrodes that they can place without requiring technicians. This means they can easily be deployed and used in both in-hospital and ambulatory settings. However, the trade-off for the ECG-derived RR estimation algorithms is between accuracy and complexity. While the more efficient algorithms that have been developed for RR estimation from surface ECG waveforms require multi-lead data, others need extraction of complex time and frequency domain features for precise RR estimation. On the other hand, overly simplified algorithms may not always provide accurate RR information. We have implemented a solution that combines the simplicity of single-lead estimation with the accuracy of multi-domain feature extraction. Furthermore, our algorithm is scalable and adaptable for different patient populations and real-time ambulatory monitoring. We developed the code using primarily open-source Python packages, which enables the code to be transferred to different platforms. Based on the application, different parameter settings in the algorithm can be adjusted to increase computational efficiency or improve accuracy.

ECG-derived RR estimation is based on the morphological properties of the ECG, which are influenced not only by respiratory patterns but also by the underlying cardiac physiology. Hence, changes in lung impedance due to respiratory disorders, or the onset of cardiac electrical abnormalities, might alter ECG morphology, affecting RR estimation. Previous studies have compared the efficacy of ECG-derived RR algorithms based on extracting different features of the beat morphology^34,35^. Yet, a thorough assessment of the efficacy of these algorithms under cardiac disease conditions has not been reported^36^. In this study, we performed a comprehensive evaluation of RR estimation under varying physiological conditions associated with exercise and changing ECG morphology with the progression of MI. The feasibility of single-lead ECG-derived RR was first tested in spontaneously breathing humans while performing different tasks. The algorithm's robustness was then validated in a controlled setting using mechanically ventilated sheep breathing at predefined constant RRs. This work demonstrates the algorithm's efficacy in estimating RR during various activities, RRs, and cardiac physiological conditions. In future clinical studies, a rigorous evaluation of the algorithm in mechanically ventilated patients could be performed to test the algorithm's accuracy in a larger cohort and assess the effect of age, gender, body mass index, and disease progression on ECG-derived RR estimation.

Our data demonstrate that accurate RR estimation is possible using single-lead ECG with a clinically acceptable error of less than one breath per minute. The location of the lead did not significantly affect the algorithm performance since the human data were acquired using the Hexoskin recorder with sensors placed near the abdomen and thorax, while the ovine data was acquired from electrodes placed near the upper arms. However, a more detailed study would need to be performed to statistically compare the algorithm accuracy based on electrode location. The algorithm's performance can be improved further by adding more accurate R-peak detection algorithms and sophisticated QRS identifiers to detect and replace erroneous or abnormal heartbeats automatically. In addition, the algorithm can be combined with predictive artificial intelligence models to accurately identify impending episodes of apnea or cardio-respiratory distress based on RR changes^37–40^.

### Limitations

The current study has some limitations. The algorithm was developed and tested using a limited dataset of healthy adults. This is due to the lack of availability of larger open-source databases with reference RR for comparison. However, the range of RRs tested in our work covered a wide range of breathing rates in the subjects, from 5–40 bpm. We did not test the effect of age, sex, and body mass on the algorithm performance; the database employed in the human study did not report this information. It is possible that subjects' body mass index might affect lung ventilation capacity and, in turn, influence the cardiac axis. Hence, additional studies would be required to test the algorithm's efficacy in subjects with differing body habiti. Because assessing respiratory function in pediatric patients is also important, additional work will need to be performed testing the algorithm in different age groups, especially newborns and infants, which have faster heart and RRs. Yet, it is possible that this simplified, computationally efficient, single-lead RR-estimator might be easily scalable and adaptable for different patient populations. Moreover, the algorithm was not tested in a real-time remote health monitoring application. It is anticipated that it will be integrated into a connectable ECG monitoring device for this application.

## Methods

### Algorithm design and Python implementation

We generated a simplified, single-lead ECG-based RR estimation algorithm that quantifies changes in the amplitude of ECG waveform R-peaks. This algorithm detects oscillations in the RMS of a moving window of individual QRS complexes, partly inspired by a previous report by Weiss et al.^26^. However, significant modifications were introduced in our approach to enhance its utility. For example, we did not include an iterative analysis of ECG data streams obtained from multiple ECG sensor leads. Instead, we optimized an algorithm so that it estimates RR by analyzing the features of QRS signals from a single lead.

**Figure 5:**
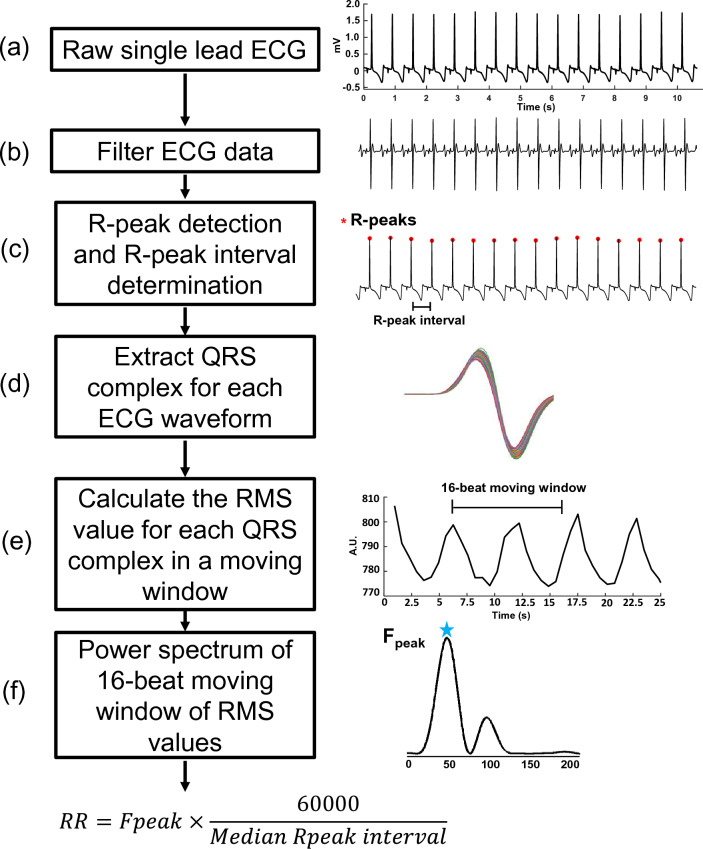
Block diagram of respiration rate estimation algorithm. Raw single-lead ECG data are filtered (panels **a** and **b**), R-peaks are detected (red symbols, panel **c**), R-peak intervals are determined, QRS complexes are extracted (panel **d**) and their root mean square amplitude (RMS) values are calculated (panel **e**), a power spectrum is generated for a moving window of 16 QRS RMS values incremented one value at a time (panel **f**), and its peak frequency and the R-peak interval data within the window are used to calculate the respiratory rate (RR) using the equation shown.

A block diagram of the algorithm is presented in Fig. 5, together with illustrative ECG waveforms. A single-lead stream of ECG data is first filtered to eliminate noise and other artifacts using an algorithmic band pass filter and methods optimized by others for ECG signal processing^41^. Next, the ECG signal peaks associated with R-waves (R-peaks) are detected using a two-averaging method^41^. Subsequently, the intervals between the R-peaks are determined, individual QRS complexes within an 80ms window centered on the R-peaks are extracted, and the root mean square amplitude (RMS) of each QRS complex is calculated. A power spectrum is then generated for overlapping moving windows of 16-RMS values, incremented one value at a time, using a 512-point Fourier transform. During the algorithm optimization, different moving window sizes of 8, 16, 32 and 64 RMS values were tested for RR estimation. The 16-beat window was chosen for the final algorithm as it provided the most optimal results. It prevents the over-smoothing of the data, caused by the larger moving windows, and enables better resolution than the 8-beat window, providing more accurate power spectrums for RR extraction. Next, the algorithm identifies the dominant power spectral peak in a frequency range that is defined by the user, corresponding to the anticipated RR range. The user can choose to employ a default estimated RR range of 1.8–18 bpm (0.03–0.3 Hz), shown to be accurate in previous studies^42,43^. However, given the wider anticipated RR range in the current study, a range of 5–40 bpm was used. The RR is then determined using the equation shown in the figure. Finally, to account for erroneous fluctuations in RR, data smoothing is performed using the median value over a moving window of 16 RR determinations to generate the final RR estimates.

The algorithm is coded in Python and available on the online project repository^44^. The open-source nature of the Python script, which employs freely available modules and well-documented code, allows the algorithm to be easily tested or modified for future applications and use. For example, the interface encountered when executing the script permits the user to test it using demonstration data provided in the online project repository or their own data after specifying the ECG sampling frequency and binary encoding method. Moreover, by entering the anticipated RR range, the window size of the smoothing function employed by the script on the data will help compensate for the signal quality of the user’s ECG-obtaining instrumentation. The algorithm was written using an integrated development environment (PyCharm Community Edition, JetBrains) and interpreted using Python 3.11. Open-source packages were leveraged to enable ease of reproducibility and transferability across platforms. The ‘numpy’ module was used to read the ECG data file, handle data, and perform mathematical operations. The script is set to read ECG data in 16-bit binary format. However, with small changes in the code by the user, it is possible that single-lead ECG data can be imported for analysis in other bit-depths and formats, such as text, matlab, or csv files. The ‘scipy’ signal processing library was used to generate filters and implement Fourier transform functions. We utilized the two-averaging R-peak detection method because it has been shown to be robust^41^. Briefly, the method incorporates a bandpass filter of 8–40 Hz to isolate QRS complexes based on the dual moving average method; a first moving average window is applied to the filtered ECG to identify the QRS intervals. A second moving window is used to identify the duration of the beat, and finally, segments where the QRS window magnitude is larger than the beat window magnitude are extracted to identify and refine R-peaks. However, the open-source nature of our work means that the user could utilize other R-peak detection algorithms^45^ for their particular application and data type. This is because different peak detection algorithms might have greater efficacy in R-peak detection based on the underlying ECG signal quality^46^. Some other commonly used R-peak detectors include the Pan-Tompkins^47^, Hamilton^48^, Christov^49^, or stationary wavelet transform^50^ detectors. Finally, the ‘matlabplotlib’ library was used to generate plots of the ECG waveforms and algorithm-determined R-peaks and RRs. The Python script (RR_estimator.py) and its interpreter and library dependency information (README.txt) are in the online project repository^44^. Also, representative single-lead ECG data from human and sheep subjects and their expected output plots (Figures S1 and S2) and csv file outputs (Table S1) are provided.

### Human subject data

The ECG and RR data used for our analysis were obtained from seven adult subjects during three levels of exercise on a treadmill. The original dataset was acquired by Vollmer and coworkers from thirteen spontaneously breathing healthy adults (seven female and six male) with an age range of 21–35 years (28 ± 4 years old) while the subjects were connected to several different physiologic recording devices and performing predetermined physical and cognitive tasks. These data reside on an online repository^28^ and are available for experimental use. For our work, we used aligned ECG and RR data cataloged on the repository obtained from the subjects using Hexoskin, a commercially available biometric shirt-like sensor assembly (Carré Technologies). This device obtains ECG signals from a single electrode. It also reports RRs that it estimates using respiratory inductive plethysmography and sensors encompassing the thorax and abdomen. The soundness of the ECG waveform data and RR estimates obtained using Hexoskin, used as reference data in the current study, have been validated previously by other groups under various physiological settings^29,51^.

However, the repository ECG and RR data streams were not supplied with a clear delineation between the tasks. Moreover, inspection of the data revealed several periods when the ECG signals had significant noise and artifacts, and the RR data had abrupt and non-physiologic changes in their values. These data were probably obtained while the biometric shirt sensors moved on the subject and the sensors were not connected properly. Therefore, for our analysis, we used ECG and RR data intervals from the subset of adult subjects during which we could identify noise-free and stable signals. We chose to study the accuracy of our RR-estimating algorithm during sequential exercise tasks in which the subject was: resting, standing upright on a treadmill (interval 1); walking on a treadmill at a moderate speed of 1.2 m/s (interval 2); or walking at the moderate speed on the treadmill at a 15% incline (interval 3). Each subject-task interval was treated as an independent value. The ECG signals had been acquired at 256Hz; for our analysis, we resampled the ECG signals at 1000Hz to ensure the fidelity of the signal waveform.

### Animal model

Myocardial ischemia and infarction induce changes in the ECG morphology that might alter the QRS power spectrum and affect the ability of the algorithm to estimate RR. To investigate the accuracy of RR estimation during ischemic heart disease, we utilized our previously established ovine model that is known to recreate the chronic MI phenotype observed in patients^30^. The nature of this model would allow us to study the effect of progressive changes in single-lead ECG QRS morphology at three time-points associated with heart disease—pre-intervention baseline, myocardial ischemia just after coronary occlusion, and MI after chronic coronary occlusion—on algorithmic RR estimation. Moreover, the mechanical ventilation of the anesthetized sheep used in this study would allow us to have more precise control of ground truth RR and to be able to alter it rapidly to test the responsiveness of the algorithm estimations. These kinds of data cannot be obtained using human subjects.

The animal experimental protocol followed the European rules for animal experimentation (European legislation 2010/63/UE-2010), which was implemented under French legislation in February 2013, reviewed and approved by the local Ethics Committee "Comité d'Ethique en Expérimentation Animale de Bordeaux”—CEEA50, and reported following the ARRIVE guidelines. The study included 18 male or female adult sheep (13 MI and 5 sham), weighing 40–60 kg. The experiments were performed using body surface ECG signals obtained using our previously described sheep MI model^30,52,53^. Briefly, after inducing anesthesia using 2 mg/Kg IV propofol, the sheep were intratracheally intubated and then mechanically ventilated using a volume-controlled ventilator (Carestation 650, GE Healthcare) at a RR of 12 bpm while they were maintained under anesthesia using 2% isoflurane in 50% O_2_ (by volume) and continuously monitored using pulse oximetry. The animals were then percutaneously catheterized via the right femoral artery, targeting the left coronary ostia using guidewires and JL4 Cordis diagnostic catheters for coronary radio-angiographic guidance under fluoroscopy. A 0.018″ microcatheter (Boston Scientific), directed by a 0.016″ steerable guidewire (Boston Scientific), was positioned in the first medial branch of the left anterior descending artery. A single 3 mm embolization coil coated with nylon fibers (VortX Diamond Coil, Boston Scientific) was deployed in the target coronary vessel. Coronary flow was determined at the level of the embolization coil before, during, and after the deployment of the embolization coil was verified by fluoroscopy using a brief injection of contrast agent (Iobitridol 658 mg/ml) until complete vascular occlusion was achieved. For the sham group, a similar intervention was performed without the coil deployment. Single-lead body surface ECG signals were obtained during the procedure and 90 min after coil placement using the AD Instruments PowerLab 4/35 system with Labchart 8 software. ECG was acquired at a sampling rate of 4kHz with the PowerLab bio-amplifier equipped with a 0.5–150 Hz bandpass filter and 50 Hz line filter. The electrode was placed near the upper arms or the thorax, with a reference electrode placed on the right leg.

After completion of the procedure, animals were extubated and transferred to a postoperative care facility for six weeks. At the end of that time, surface electrodes were placed again on animals after they were anesthetized, intubated, and mechanically ventilated, and a follow-up ECG measurement was performed, as described above. At the completion of the study, the animal was euthanized using 80 mg/kg IV pentobarbital.

### Data and statistical analysis

For the spontaneously breathing humans, RRs were estimated during the execution of tasks. A cycle-to-cycle comparison of the estimated RR and the expected reference RR was performed during each evaluated intervention. For the mechanically ventilated sheep, RRs were estimated from ECG acquired at baseline, 30 min after coronary artery occlusion (ischemia), and six weeks post-development of chronic MI. The RRs were then compared with the reference ventilator RR at the three-time points in both sham and MI groups defined above. In one sham animal, the ventilator RR was changed according to a predefined pattern of 11 bpm, 10 bpm, 12 bpm, 10 bpm, and then 11 bpm with ~ 2 min of ECG data collected at each RR setting.

All categorical data are presented as mean ± standard deviation. The efficacy and precision of the algorithm in estimating RR and distinguishing between different settings were evaluated. The correlation between instantaneous estimated and expected RRs was evaluated using a linear fit model and the absolute and relative error in RR estimation during each subject-task intervention was evaluated. The data are presented as boxplots with median (horizontal solid line), 75–25% percentiles (box), and error bars. A statistical equivalence test was used to determine whether the estimated and expected RR were similar. For this analysis, two one-sided tests (XLSTAT) were employed with a ± 2 bpm threshold chosen for a 90% confidence interval of the significance. For the sheep study, the RR distributions at the different ventilator rates were compared using the non-parametric Wilcoxon rank sum test. A *p*-value < 0.05 was considered statistically significant. Statistical analysis and data presentation were performed using MATLAB 2020b (MathWorks Inc, Natick, MA) software.

### Supplementary Information

Supplementary Information 1.

Supplementary Information 2.

## Data Availability

The human subjects’ data are open-source and available on Physionet^28,29,54^. The animal subjects’ data used in the analysis are available upon request from Dr. Kulkarni.
