# Supplementary material for: Open-source software for respiratory rate estimation using single-lead electrocardiograms
Source: Sci Rep. 2024 Jan 2;14:167. doi: 10.1038/s41598-023-50470-0 (PMC10762020; doi:10.1038/s41598-023-50470-0)
Supplement: Supplementary file 1 — Supplementary Information 1. [file 41598_2023_50470_MOESM1_ESM.pptx]

## Slide 1
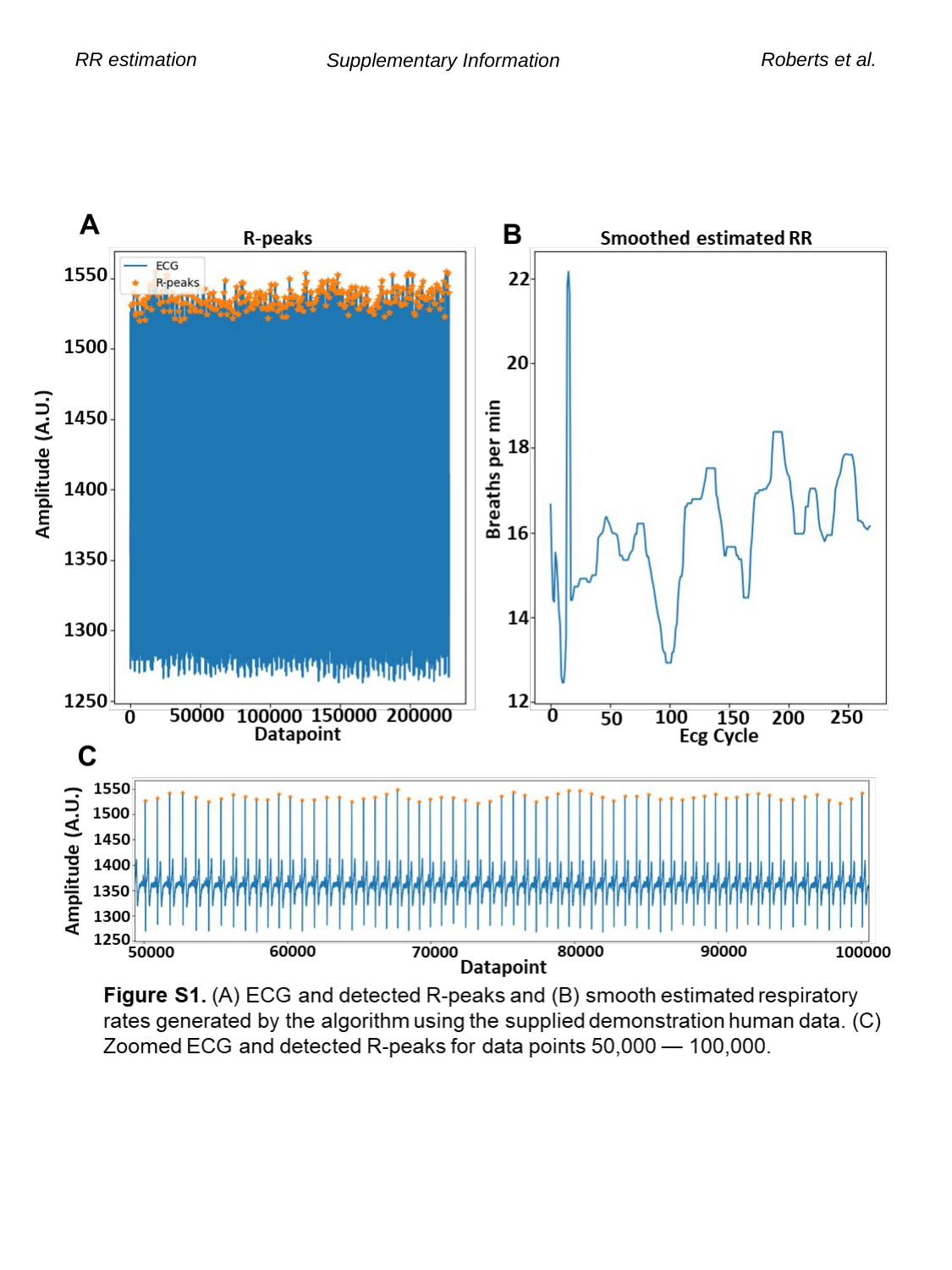

RR estimation
Roberts et al.
Supplementary Information

## Slide 2
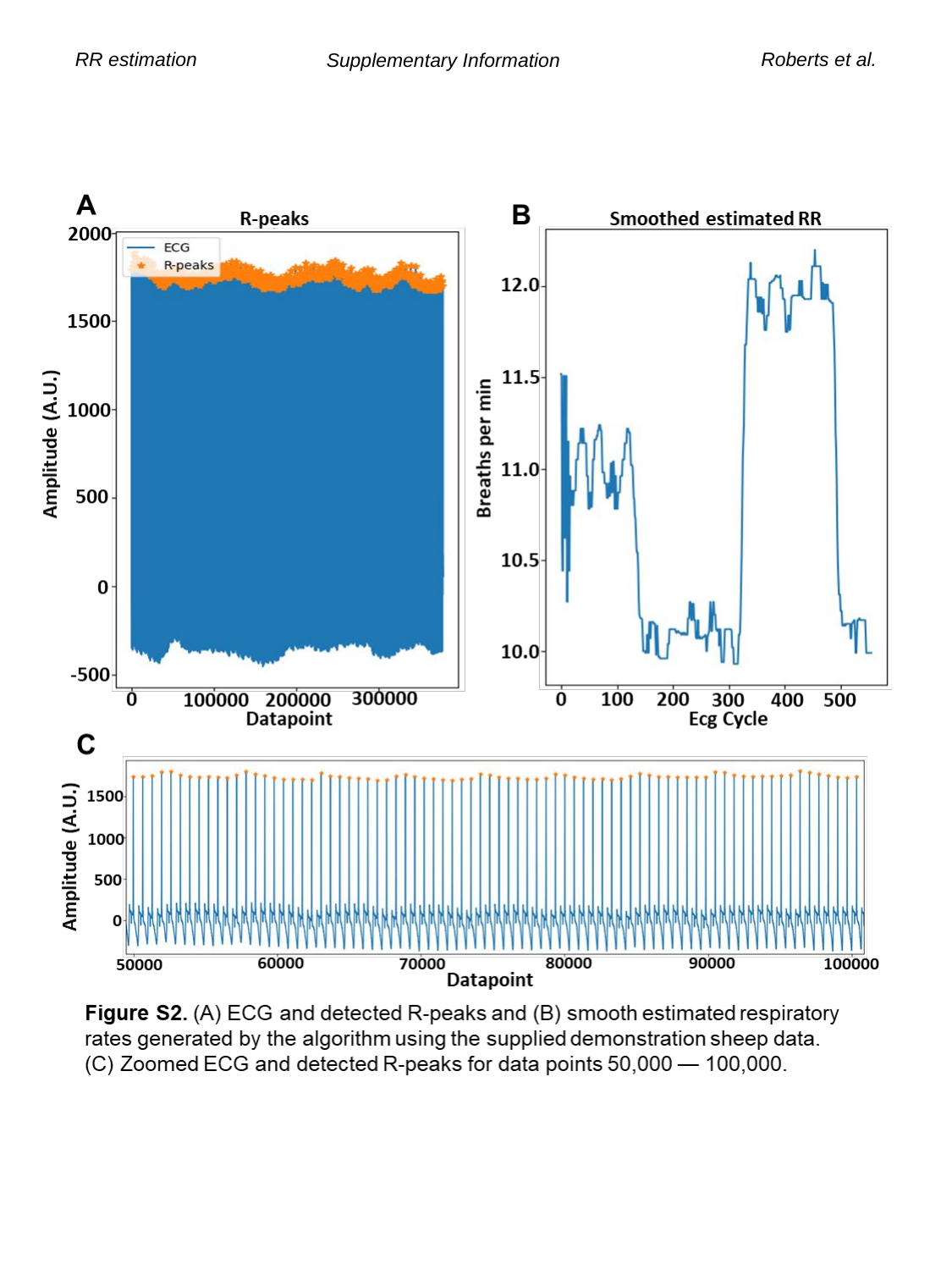

RR estimation
Roberts et al.
Supplementary Information

## Slide 3
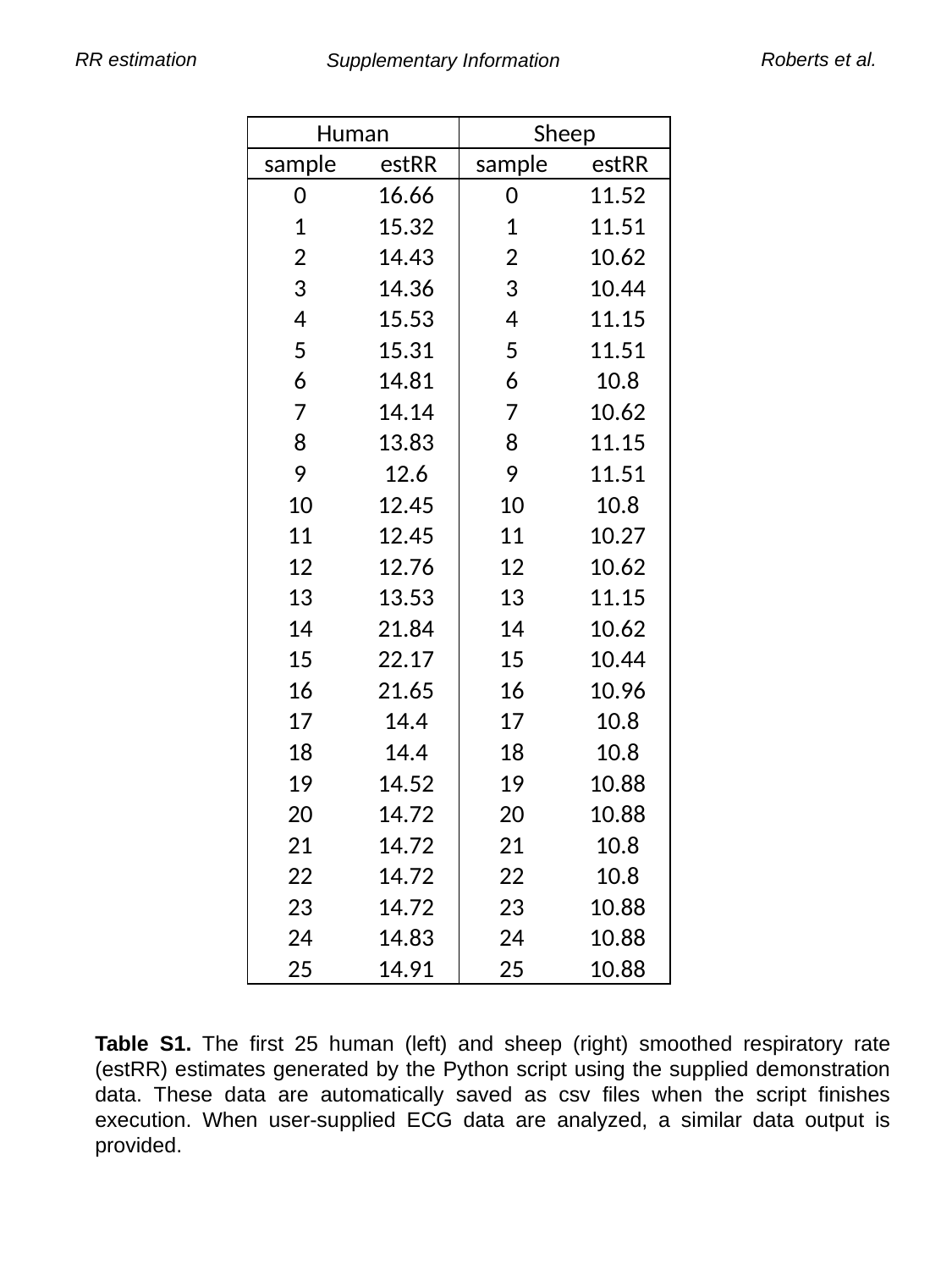

RR estimation
Roberts et al.
Supplementary Information
| Human | | Sheep | |
| --- | --- | --- | --- |
| sample | estRR | sample | estRR |
| 0 | 16.66 | 0 | 11.52 |
| 1 | 15.32 | 1 | 11.51 |
| 2 | 14.43 | 2 | 10.62 |
| 3 | 14.36 | 3 | 10.44 |
| 4 | 15.53 | 4 | 11.15 |
| 5 | 15.31 | 5 | 11.51 |
| 6 | 14.81 | 6 | 10.8 |
| 7 | 14.14 | 7 | 10.62 |
| 8 | 13.83 | 8 | 11.15 |
| 9 | 12.6 | 9 | 11.51 |
| 10 | 12.45 | 10 | 10.8 |
| 11 | 12.45 | 11 | 10.27 |
| 12 | 12.76 | 12 | 10.62 |
| 13 | 13.53 | 13 | 11.15 |
| 14 | 21.84 | 14 | 10.62 |
| 15 | 22.17 | 15 | 10.44 |
| 16 | 21.65 | 16 | 10.96 |
| 17 | 14.4 | 17 | 10.8 |
| 18 | 14.4 | 18 | 10.8 |
| 19 | 14.52 | 19 | 10.88 |
| 20 | 14.72 | 20 | 10.88 |
| 21 | 14.72 | 21 | 10.8 |
| 22 | 14.72 | 22 | 10.8 |
| 23 | 14.72 | 23 | 10.88 |
| 24 | 14.83 | 24 | 10.88 |
| 25 | 14.91 | 25 | 10.88 |
Table S1. The first 25 human (left) and sheep (right) smoothed respiratory rate (estRR) estimates generated by the Python script using the supplied demonstration data. These data are automatically saved as csv files when the script finishes execution. When user-supplied ECG data are analyzed, a similar data output is provided.
